# Monitoring and Early Warning of the Cage-Rearing Broiler Farming Environment Based on an Inspection Robot

**DOI:** 10.3390/ani16091417

**Published:** 2026-05-06

**Authors:** Sai Luo, Xiangchao Kong, Xintong Xie, Wanchao Zhang, Pengshen Zheng, He Zhu, Deqi Hao, Jingkun Sun, Changxi Chen

**Affiliations:** 1 Key Laboratory of Smart Breeding (Co-Construction by Ministry and Province), Ministry of Agriculture and Rural Affairs, Tianjin 300384, China; luosai_678@163.com (S.L.); kxc_ouc@163.com (X.K.); xxt17u@163.com (X.X.); 2 College of Computer and Information Engineering, Tianjin Agricultural University, Tianjin 300384, China; zhangwanchao@tjau.edu.cn (W.Z.); zhuhe_2078@163.com (H.Z.); haodeqi_3178@163.com (D.H.); ekko201@163.com (J.S.)

**Keywords:** inspection robot, cage-reared broiler house, temperature deviation, temperature–humidity index (THI), wavelet decomposition, gated recurrent unit (GRU), efficient channel attention (ECA)

## Abstract

In large-scale caged broiler houses, the combined effects of ventilation regulation, external meteorological disturbances, and changes in flock growth stages can easily cause the indoor thermal environment to deviate from the target settings, thereby increasing the difficulty of environmental control and production risks. Meanwhile, the broiler house environment exhibits pronounced spatial heterogeneity, while manual inspection is limited by low efficiency, high labor intensity, and delayed detection of anomalies. Therefore, this study proposes an inspection-robot-based method for predicting and providing early warnings for temperature deviation (TD) and temperature–humidity index (THI). In this method, TD is used to characterize the degree of deviation in environmental control, whereas THI is used to characterize the overall thermal environmental risk under the coupled effects of temperature and humidity. For the two prediction tasks, input variables were separately selected based on correlation analysis, and the same Wavelet–ECA–GRU model architecture was adopted for training to achieve short-term prediction over the next 30 min. The results show that the proposed model outperformed baseline models, including LSTM, TCN, and GRU. On the test set, the RMSE and R^2^ for TD prediction were 0.2886 and 0.9238, respectively, while those for THI prediction were 0.4309 and 0.9287, respectively. Based on the TD classification results and THI risk thresholds, warning strategies for heat deviation, cold deviation, and humid-heat risk were established. The proposed method can identify potential thermal environmental anomalies in caged broiler houses in advance from the perspectives of environmental control deviation and comprehensive thermal environmental risk, thereby assisting farm managers in assessing whether timely adjustments to ventilation, heating, or cooling are needed.

## Simple Summary

Environmental conditions often vary markedly across different locations within caged broiler houses. Relying solely on pre-installed fixed monitoring points makes it difficult to detect potential thermal environmental risks in a timely manner. To address this issue, this study developed an integrated monitoring system consisting of an inspection robot and fixed monitoring points to enhance environmental monitoring capacity in broiler houses and enable early identification of potential environmental anomalies. The system can help farm managers detect emerging thermal environmental problems at an earlier stage and, in combination with field observations of flock status, determine whether management interventions such as adjustments to ventilation, heating, or spraying are required. This approach can improve the timeliness of environmental regulation in broiler houses and support broiler health and welfare.

## 1. Introduction

The thermal environment is a key factor affecting broiler production performance, health status, and animal welfare. Modern broilers are characterized by rapid growth, high metabolic rates, and high heat production per unit body weight, making them particularly sensitive to adverse environmental conditions such as high temperature and humidity. When environmental control in broiler houses is inappropriate, broilers may experience reduced feed intake, inhibited weight gain, elevated body temperature, increased respiratory rate, and impaired immune function; in severe cases, the risk of mortality and culling may also increase [1,2]. Therefore, timely and accurate perception of broiler house environmental conditions, together with risk warnings before abnormalities develop, is of great significance for ensuring healthy broiler production, improving animal welfare, and enhancing environmental regulation [3,4,5].

In studies evaluating the thermal environment of livestock and poultry housing, the temperature–humidity index (THI) has been widely used in heat stress research because it can comprehensively characterize thermal load under the coupled effects of temperature and humidity [6,7]. Previous studies have shown that THI is closely associated with physiological responses, changes in production performance, and heat stress status in broilers; therefore, it has become one of the important indicators for assessing thermal environmental risk in broiler production. In addition to THI, comprehensive thermal environmental indices such as apparent temperature have also been used to characterize the actual environmental load experienced by broiler flocks [8]. Compared with single environmental parameters, these indices can, to some extent, comprehensively reflect the combined effects of temperature, humidity, and other environmental factors on the thermal environmental status of broilers, and thus have high application value in evaluating the thermal environment of broiler houses.

During environmental control in broiler houses, the degree to which the actual temperature deviates from the target temperature is also worthy of attention. Previous studies have evaluated broiler house thermal environments and control performance from the perspective of deviations between actual environmental temperature and target temperature, or between actual values and set points [9]. Unlike THI, which focuses on assessing thermal environmental risk under the coupled effects of temperature and humidity, temperature deviation is more suitable for characterizing the extent to which environmental regulation deviates from the preset target. In addition to the above indicators, some studies have also comprehensively evaluated broiler house environmental conditions from the perspectives of environmental suitability and discomfort, providing a more intuitive basis for environmental quality assessment and management decision-making [10,11].

The accurate assessment of environmental conditions largely depends on whether monitoring data can adequately represent the actual environment experienced by broiler flocks. At present, environmental monitoring in broiler houses is still mainly based on fixed-point sensors, which collect continuous data through the deployment of devices for measuring temperature, humidity, gas concentrations, and other parameters [12,13]. This approach is convenient for installation and long-term operation. However, the internal environment of a broiler house is not uniformly distributed, and microenvironmental conditions often differ among regions, heights, and flock density levels. Therefore, fixed-point monitoring results may not fully reflect the actual environment to which broilers are exposed. In recent years, mobile robots and intelligent inspection technologies have begun to be introduced into environmental monitoring in livestock and poultry houses. Compared with fixed high-position monitoring, mobile inspection enables dynamic sampling in the activity areas of broilers, thereby obtaining environmental information with broader coverage and stronger spatial representativeness [14]. Nevertheless, existing studies have mainly focused on equipment development and exploratory applications of environmental monitoring, while how to predict key environmental indicators and identify risks based on inspection data remains insufficiently investigated [15].

From a modeling perspective, environmental data in broiler houses usually exhibit strong non-stationarity, obvious local fluctuations, and short-term disturbances. They are also affected by the coupled effects of multiple environmental factors, which poses considerable challenges for environmental time-series prediction. Previous studies in the fields of agriculture and livestock housing environments have applied deep learning methods such as RNN, LSTM, and GRU to predict environmental parameters, including temperature, humidity, and gas concentrations, and have achieved favorable results [16,17]. On this basis, some studies have further combined sequence decomposition methods with neural networks to improve the ability of models to characterize complex sequences by extracting variation information at different scales [18,19]. Other studies have introduced attention mechanisms to weight key information in multivariate inputs, thereby improving prediction accuracy [20,21,22]. However, most existing studies are still based on fixed-point monitoring data, and their prediction targets mainly focus on a single environmental variable or a single comprehensive index. Meanwhile, studies related to mobile inspection have placed greater emphasis on equipment development and exploratory applications in environmental monitoring. Compared with the most closely related studies, the present study focuses on inspection scenarios in caged broiler houses, separately models two indicators—temperature deviation (TD) and temperature–humidity index (THI)—and further establishes a joint early-warning strategy to identify broiler house environmental conditions from the perspectives of both environmental control deviation and thermal environmental risk.

Based on the above considerations, this study developed an application-oriented monitoring–prediction–warning framework for caged broiler houses under inspection-based monitoring scenarios, integrating environmental perception, TD/THI short-term prediction, and dual-indicator early warning for the early identification of thermal environmental anomalies. In this framework, inspection-based monitoring is used to improve the spatial representativeness of environmental perception; TD characterizes the deviation of the actual indoor temperature from the target temperature and reflects environmental control deviation; and THI characterizes thermal environmental risk under the combined effects of temperature and humidity. Compared with prediction studies that focus only on a single environmental parameter or a single thermal environmental indicator, this study further uses the TD and THI prediction results for warnings of heat deviation, cold deviation, and humid-heat risk, thereby enabling the early identification of thermal environmental anomalies in broiler houses. When warning information is issued by the system, farm managers can combine the warning results with field observations of flock status, such as feed intake, water intake, activity level, and respiratory condition, to comprehensively assess thermal environmental risk and accordingly adjust ventilation rate, heating intensity, and cooling measures in a timely manner.

## 2. Materials and Methods

### 2.1. Data Sources and Acquisition System

The experiment was carried out in a commercial poultry farm located in Dezhou, Shandong Province, China, from 26 May to 29 June 2025, spanning one complete broiler production cycle of approximately 5 weeks. The tested house accommodated more than 30,000 birds. Structurally, it was a standardized elongated cage-rearing house, measuring about 101 m in length and 16 m in width, with an eave height of approximately 4.2 m and a ridge height of approximately 6 m. Inside the house, cages were installed in a longitudinal arrangement and organized into seven independent rows. Each row consisted of four tiers, and each tier contained about 73 cage positions. Individual cages measured approximately 1.25 m × 1.0 m × 0.45 m (length × width × height).

**Figure 1:**
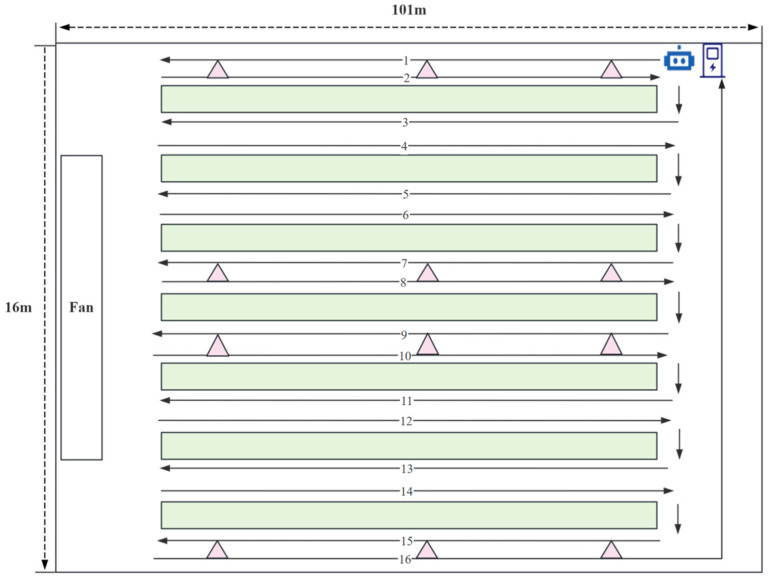
Schematic diagram of the internal layout of the experimental broiler house, the distribution of fixed monitoring points, and the inspection route of the inspection robot.

To achieve continuous monitoring and dynamic perception of the broiler house environment, this study established a multi-source environmental data acquisition system consisting of an inspection robot, fixed indoor monitoring points, and an outdoor monitoring point. All the above devices were developed in-house by Tianjin Agricultural University (Tianjin, China). As shown in Figure 1, the green rectangles represent the chicken cages, channel numbers 1–16 indicate the inspection route of the robot, the arrows indicate examples of the inspection direction, and the purple triangles represent the fixed monitoring points arranged inside the broiler house. A total of 12 fixed monitoring points were installed inside the broiler house, located in Channels 1, 4, 5, and 8. In each selected channel, one monitoring point was placed at the front, middle, and rear sections along the longitudinal direction of the house. The inspection robot was mainly used to obtain dynamic environmental information from the broiler activity area, thereby improving the spatial representativeness of environmental perception in the broiler house. The fixed indoor monitoring points were mainly used to analyze environmental differences along both the longitudinal and lateral directions of the house, reflecting the overall spatial distribution characteristics of the indoor environment. The outdoor monitoring point was used to synchronously collect outdoor environmental parameters, providing basic data for analyzing the relationship between indoor and outdoor environmental changes. During the experiment, the inspection robot conducted scheduled inspections along a preset route in key areas of the broiler house. The inspection speed was set to 0.3 m/s, and one complete full-route inspection required approximately 96 min.

**Figure 2:**
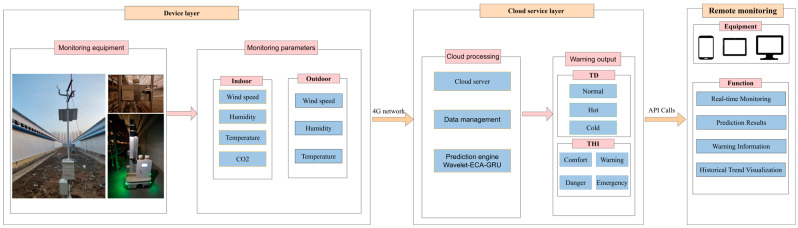
Architecture of the robot-based poultry house environmental monitoring, prediction, and early-warning system.

As shown in Figure 2, multi-source environmental monitoring data were uploaded in real time to a remote server via a wireless network, where data storage, timestamp alignment, state identification, preprocessing, resampling, prediction analysis, and early-warning information generation were performed. The inspection robot recorded indoor environmental data, including temperature, relative humidity, air velocity, and carbon dioxide concentration, at a sampling interval of 3 s. The fixed indoor monitoring points continuously and synchronously collected the corresponding environmental parameters, while the outdoor monitoring point continuously collected outdoor environmental parameters, including temperature, relative humidity, and air velocity. After completing each inspection task, the inspection robot automatically returned to the charging dock for recharging and waited for the next scheduled task. During the inspection interval and charging waiting period, the environmental monitoring devices mounted on the robot remained in operation. To ensure temporal consistency among environmental information from different sources, all monitoring terminals attached timestamps during data acquisition. After the data were uploaded to the server, they were sorted and aligned according to a unified time reference. During the alignment process, a basic temporal resolution of 3 s was used to map the data from the inspection robot, fixed indoor monitoring points, and outdoor monitoring point onto a unified time scale. When slight deviations existed among the recording times of different devices, nearest-neighbor time matching was adopted to merge the data into the same time point. After time alignment, the time-aligned 3 s data were resampled and aggregated using a 5 min time window. Within each 5 min window, valid records of each environmental variable were averaged to obtain the representative value for that interval. Invalid records, such as values beyond the sensor measurement range or duplicate records, were excluded before aggregation. If no valid value was available for a given variable within a 5 min window, the corresponding aggregated value was marked as missing and then handled according to the missing-value procedure described in Section 2.2. This aggregation process reduced the influence of instantaneous noise and maintained consistency with the time step of the prediction model. The preprocessed time series were then input into the prediction model for real-time computation, and future environmental risk warning information was generated based on the prediction results. Finally, real-time monitoring data, alarm results, prediction results, and early-warning information were synchronously transmitted to the monitoring platform for displaying the environmental status of the broiler house and information on abnormal risks. The main sensor module models, measurement ranges, and accuracies of the monitoring terminals used in this study are presented in Table 1.

### 2.2. Data Preprocessing

The time-series data collected during the experiment were used to train the prediction models for temperature deviation (TD) and temperature–humidity index (THI) in the broiler house. Temperature deviation (TD) was defined as the difference between the measured indoor temperature and the target temperature, and its expression is given in Equation (1).

(1) $${\mathrm{TD}}_{t}=T_{\mathrm{in},t}-T_{\mathrm{target},t}$$

where $T_{\mathrm{in},t}$ is the measured indoor temperature at time t, and $T_{\mathrm{target},t}$ is the target temperature corresponding to time t. The temperature–humidity index (THI) was used to characterize the overall thermal load under the coupled effects of temperature and humidity, and its calculation formula is given in [23].

(2) THI=(1.8×T+32)−[(0.55−0.0055×RH)×(1.8×T−26)]

where *T* is the dry-bulb temperature and RH is the relative humidity. To avoid information leakage in time-series modeling, the dataset was divided into a training set and a test set at a ratio of 8:2, and feature extraction and normalization were then performed on this basis. The raw data before preprocessing contained a total of 8458 records. Missing values occurred only in the ${\mathrm{CO}}_{2}$ variable, with 191 missing values, accounting for 2.26% of the records for this variable. In this study, linear interpolation was used to impute the missing values. After this processing, no missing records remained in the dataset; therefore, no sample rows were removed.

To eliminate the differences in feature scales and improve model training efficiency, the min–max normalization method was adopted to map the input features to the interval [0, 1], as expressed in Equation (3).

(3) $$z=\frac{x-x_{\min}}{x_{\max}-x_{\min}}$$

where *x* is the original feature value, $x_{\min}$ and $x_{\max}$ were obtained from the statistics of the training set and then used for the unified transformation of both the training set and the test set. The target variables, TD and THI, were also scaled using normalizers fitted only on the training set, thereby avoiding information leakage from the test set.

Finally, supervised learning samples were constructed using a sliding-window strategy. Specifically, a multivariate sequence of the previous *L* = 36 time steps (5 min per step, totaling 180 min) was used as the input to predict the temperature deviation and temperature–humidity index at the future *H* = 6 time steps ahead (i.e., a 30-min-ahead prediction). The 30 min prediction horizon was mainly determined based on the practical requirements of environmental regulation in broiler houses. It can provide farm managers with sufficient response time to promptly implement control measures such as ventilation, heating, or spraying. If the prediction horizon is too short, the advance warning time would be limited; however, an excessively long prediction horizon would increase the uncertainty of short-term prediction. The input window was set to 180 min (*L* = 36), mainly because changes in the thermal environment of broiler houses are jointly affected by ventilation regulation, external disturbances, and heat production from broiler flocks, and therefore exhibit certain short-term lag effects and thermal inertia. Compared with a shorter window, a 180 min input window can provide more sufficient historical information to characterize local fluctuations, variation trends, and the lagged effects of control responses. In contrast, an excessively long window may introduce historical information that is weakly related to the current short-term prediction task, thereby increasing model complexity and redundant noise. Therefore, in this study, 180 min was used as the input window to balance the dynamic characteristics of the broiler house environment and the requirements of short-term prediction. The window was moved along the time series with a step size of one time step (5 min), thereby generating overlapping samples. This strategy transformed a single long time series into a large number of supervised learning samples, thus expanding the training sample size and improving the generalization ability of the model. At the same time, it preserved local temporal dependencies and control lag effects, which helped the model learn the short-term dynamic variation patterns of temperature deviation. In addition, the rolling prediction method with a step size of 1 was consistent with the online deployment scenario, facilitating real-time rolling prediction and early warning.

### 2.3. Model Construction

#### 2.3.1. Overall Model Architecture

**Figure 3:**
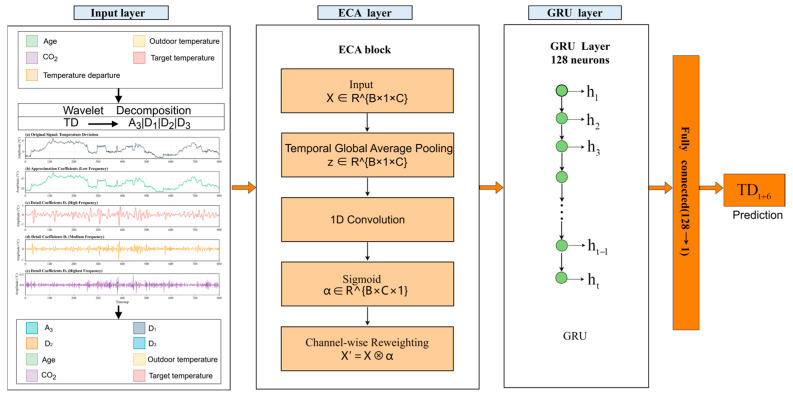
Structure of the Wavelet–ECA–GRU model, with the temperature deviation (TD) prediction task as an example.

This study adopted a Wavelet–ECA–GRU hybrid framework for the short-term prediction of temperature deviation (TD) and temperature–humidity index (THI) in broiler houses, and its overall structure is shown in Figure 3. For each prediction task, the historical series of the target variable was first decomposed at multiple scales using a sliding-window-based discrete wavelet transform (DWT) to address its non-stationary fluctuation characteristics. This process extracted the trend component and detail components at different frequency bands, thereby enhancing the model’s ability to characterize slow drift and short-term disturbances. Subsequently, the multi-scale features of the target variable obtained by wavelet decomposition were concatenated with the remaining input variables to form the model input sequence.

At the feature-weighting stage, an efficient channel attention (ECA) mechanism was introduced. Based on the statistical information of each input channel within the time window, this mechanism used a lightweight one-dimensional convolution along the channel dimension to model local inter-channel correlations and generate attention weights for each channel. In this way, the input features were adaptively weighted, thereby adjusting the relative contribution of different variables in the subsequent temporal modeling process.

At the temporal modeling stage, a gated recurrent unit (GRU) was employed to learn the temporal dependencies and lag effects in the input sequence, and a fully connected regression layer was used to output the predicted value of the target variable at the given forecasting horizon.

#### 2.3.2. Wavelet Decomposition

Broiler house environmental conditions are jointly affected by multiple factors, including ventilation regulation, heat exchange, external meteorological conditions, and changes in flock growth status. As a result, both temperature deviation (TD) and temperature–humidity index (THI) time series exhibit evident non-stationary characteristics. These characteristics include not only low-frequency trends that evolve gradually with environmental regulation and growth-stage changes, but also high-frequency components caused by equipment start–stop events, local disturbances, and fluctuations in the external environment. A single-scale feature is often insufficient to simultaneously characterize long-term variation trends and short-term local fluctuations, thereby limiting the ability of prediction models to learn complex dynamic processes. Therefore, this study introduced the discrete wavelet transform (DWT) to perform multi-scale decomposition of the target sequence, so as to extract low-frequency trend information and high-frequency fluctuation features at different scales, thereby enhancing the representation ability of the subsequent prediction model for non-stationary time series [24,25].

Considering that the prediction task must satisfy temporal causality, this study adopted a sliding-window wavelet decomposition strategy. Taking the TD prediction task as an example, for any time step t, only historical observations up to that time step were used to construct a window signal with a length of W. When the historical length was insufficient, left-side padding was performed by replicating boundary values to keep the window length consistent. Therefore, the historical window signal corresponding to time step t can be expressed as follows:

(4) $$S_{t}=\left[{\mathrm{TD}}_{t-W+1},{\mathrm{TD}}_{t-W+2},\ldots ,{\mathrm{TD}}_{t}\right]$$

On this basis, discrete wavelet decomposition was applied to the window signal $s_{t}$, yielding the level-*L* approximation coefficient sequence and the detail coefficient sequences at each level, i.e.,

(5) $$S_{t}\overset{\mathrm{DWT}}{\to }\left\{A_{t}^{\left(L\right)},D_{t}^{\left(1\right)},D_{t}^{\left(2\right)},\ldots ,D_{t}^{\left(L\right)}\right\}$$

where $A_{t}^{\left(L\right)}$ denotes the level-L approximation coefficient, which mainly reflects the low-frequency trend information of the sequence, and $D_{t}^{\left(j\right)}$ denotes the detail coefficient at level j, which is used to characterize the high-frequency fluctuation component at the corresponding scale. To ensure strict alignment between the extracted features and the current time step, the last coefficient from each coefficient sequence was extracted as the wavelet representation at time t, and a wavelet feature vector was constructed.

(6) $$F_{t}^{\mathrm{wav}}=\left[A_{t}^{\left(L\right)},D_{t}^{\left(1\right)},D_{t}^{\left(2\right)},\ldots ,D_{t}^{\left(L\right)}\right]$$

The extracted wavelet features were then concatenated with the other screened environmental variables and used as inputs to the subsequent ECA–GRU model. For the THI prediction task, the same sliding-window wavelet decomposition procedure was adopted, with only the decomposition target changed from the TD historical series to the THI historical series. In this study, the number of wavelet decomposition levels was set to 3, and the sliding window length was set to 56. To compare the effects of different wavelet basis functions on prediction performance, db2, db4, and sym4 were selected as candidate wavelet bases and further compared in subsequent experiments.

#### 2.3.3. ECA Channel Attention

Attention mechanisms are essentially a resource allocation strategy and have been widely applied in neural networks to enhance the representation of key information. Channel attention mechanisms can improve feature representation by modeling inter-channel dependencies. However, SE-style channel attention mechanisms usually rely on dimensionality reduction to capture channel-wise relationships, which may weaken the direct modeling of dependencies among the original channels and reduce the preservation of complete cross-channel interaction information. In contrast, ECA avoids dimensionality reduction and captures local cross-channel interactions through lightweight one-dimensional convolution, thereby improving feature recalibration efficiency while maintaining low model complexity [26,27].

**Figure 4:**
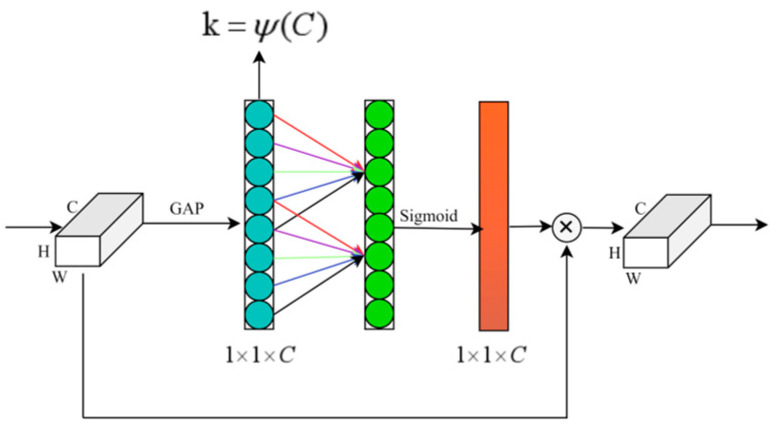
Schematic diagram of the efficient channel attention mechanism.

Figure 4 illustrates the working principle of ECA. Unlike the SE mechanism, ECA does not use fully connected layers to perform dimensionality reduction mapping of channel features. Instead, it directly models channel dependencies through a local cross-channel interaction strategy and achieves efficient channel weight allocation by means of lightweight one-dimensional convolution. To adapt to the channel interaction range under different feature dimensions, ECA adaptively selects the convolution kernel size according to the number of channels, which can be expressed as

(7) $$k=\psi (C)={\left|\frac{\log_{2}(C)+b}{\gamma }\right|}_{\mathrm{odd}}$$

Among them, C denotes the number of feature channels, b and γ are hyperparameters, and ${\left|\cdot \right|}_{\mathrm{odd}}$ indicates that the result is mapped to the nearest odd integer. Referring to the default settings of the original ECA-Net, this study fixed γ=2 and b=1, and adaptively determined the one-dimensional convolution kernel size k according to the number of channels C, without conducting an additional manual search for k. The ablation experiments in the original ECA-Net showed that adaptive convolution kernel selection generally performs better than using a fixed kernel size, while also avoiding manual parameter tuning through cross-validation [27]. Therefore, this study adopted this configuration to reduce interference caused by additional hyperparameter selection and to balance model complexity with feature representation capability.

Furthermore, ECA adaptively recalibrates the input features by assigning different weights to different feature channels. The channel weight can be expressed as

(8) $$\omega_{i}=\sigma \left(\sum_{j=1}^{k}\alpha^{j}y_{i}^{j}\right)$$

where $\omega_{i}$ denotes the attention weight of the *i*-th channel, σ(⋅) is the Sigmoid activation function, $\alpha^{j}$ denotes the shared parameters of the convolution kernel, and $y_{i}^{j}$ denotes the information response of the current channel and its local neighboring channels. Through this mechanism, channels corresponding to key variables receive higher responses, whereas redundant or strongly interfering feature channels are relatively suppressed.

#### 2.3.4. Gated Recurrent Unit (GRU)

**Figure 5:**
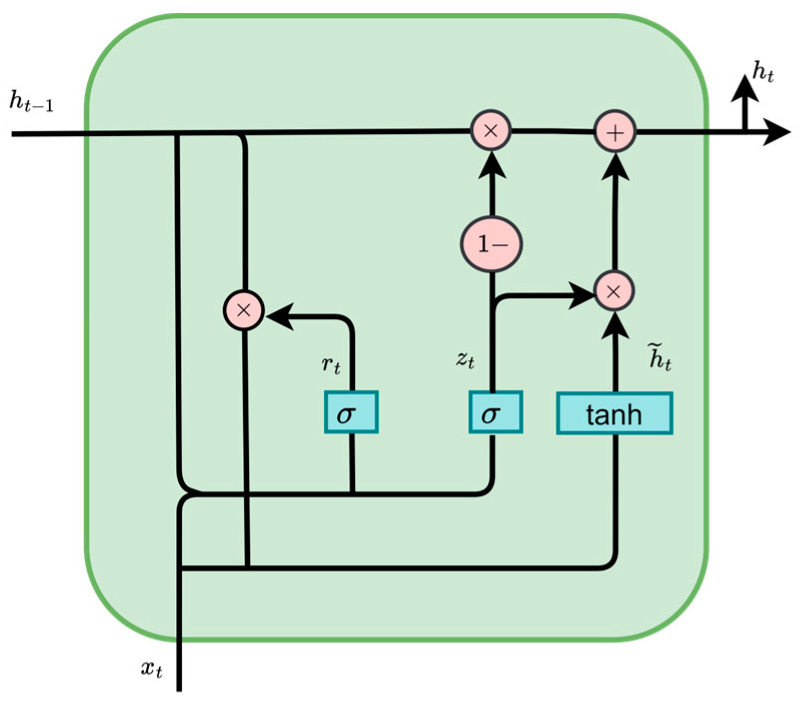
Schematic structure of the GRU.

The gated recurrent unit (GRU) is a type of recurrent neural network (RNN) with a gating mechanism. By introducing an update gate and a reset gate, GRU regulates the retention of historical information and the writing of new information, thereby alleviating the gradient vanishing problem that commonly occurs in traditional RNNs during long-sequence modeling. Compared with LSTM, GRU has a simpler structure and fewer parameters, allowing it to improve training efficiency while maintaining strong temporal modeling capability. Therefore, it is suitable for environmental sequence prediction tasks characterized by non-stationary fluctuations [28,29]. The basic structure of GRU is shown in Figure 5. At time step t, its calculation process can be expressed as follows:

(9) $$z_{t}=\sigma (W_{z}*x_{t}+U_{z}*h_{t-1})$$

(10) $$r_{t}=\sigma (W_{r}*x_{t}+U_{r}*h_{t-1})$$

(11) $${\tilde{h}}_{t}=\tanh(W*x_{t}+U*(r_{t}⊙h_{t-1}))$$

(12) $$h_{t}=(1-z_{t})⊙h_{t-1}+z_{t}⊙{\tilde{h}}_{t}$$

Among them, $x_{t}$ denotes the input feature vector at time step t, and $h_{t}$ denotes the hidden state at the current time step. $z_{t}$ and $r_{t}$ represent the update gate and reset gate, respectively, while $\tilde{h}$ denotes the candidate hidden state. W and U are learnable weight matrices, σ is the sigmoid function, and ⊙ denotes element-wise multiplication.

For an input sequence of length *L*, $\left[x_{t-L+1},\ldots ,x_{t}\right]$, the GRU outputs the corresponding hidden-state sequence $\left\{h_{t-L+1},\ldots ,h_{t}\right\}$. In this study, the hidden state at the last time step, $h_{t}$, was used as the sequence representation, and the predicted value of the target variable was output through a fully connected layer as follows:

(13) $${\hat{y}}_{t+p}=W_{o}h_{t}+b_{o}$$

where ${\hat{y}}_{t+p}$ denotes the predicted value at the future step p, and $W_{o}$ and $b_{o}$ denote the weight matrix and bias term of the output layer, respectively.

#### 2.3.5. Experimental Environment and Parameter Settings

The experimental hardware configuration included an Intel i7-14650HX CPU with a clock speed of 2.20 GHz, an RTX 4060 GPU, and 16 GB of memory. During model training, the Adam optimizer was used with a learning rate of 0.001, a batch size of 32, and a maximum of 200 training epochs, together with an early stopping strategy. The input window length was set to 36 time steps, and the prediction horizon was set to 6 time steps. The hidden dimension of the GRU layer was set to 128, and the number of network layers was set to 1. For both the TD and THI prediction tasks, three-level wavelet decomposition was adopted, and modeling and training were conducted separately under the same Wavelet–ECA–GRU framework.

#### 2.3.6. Evaluation Indicators

To quantitatively evaluate the predictive performance of different models, mean squared error (MSE), mean absolute error (MAE), root mean square error (RMSE), and the coefficient of determination ($R^{2}$) were adopted as evaluation indicators. Their calculation formulas are given in Equations (14)–(17).

(14) $$\mathrm{MSE}=\frac{1}{N}\sum_{i=1}^{N}{\left(y_{i}-{\hat{y}}_{i}\right)}^{2}$$

(15) $$\mathrm{MAE}=\frac{1}{N}\sum_{i=1}^{N}\left|y_{i}-{\hat{y}}_{i}\right|$$

(16) $$\mathrm{RMSE}=\sqrt{\frac{1}{N}\sum_{i=1}^{N}{\left(y_{i}-{\hat{y}}_{i}\right)}^{2}}$$

(17) $$R^{2}=1-\frac{\sum_{i=1}^{N}{\left(y_{i}-{\hat{y}}_{i}\right)}^{2}}{\sum_{i=1}^{N}{\left(y_{i}-\bar{y}\right)}^{2}}$$

where N denotes the sample size, $y_{i}$ denotes the actual value, ${\hat{y}}_{i}$ denotes the predicted value, and $\bar{y}$ denotes the mean of the actual values.

### 2.4. Real-Time Monitoring Module for the Rearing Environment

To improve the representativeness of broiler house environmental perception and the timeliness of anomaly identification, a real-time broiler house environmental monitoring module was developed based on the inspection robot platform.

During the operation of the monitoring module, the inspection robot and fixed environmental data acquisition devices synchronously collected environmental parameters at the preset frequency and uploaded the data to the server in real time through a wireless network. On this basis, the system incorporated broiler age information and retrieved the suitable environmental ranges corresponding to different growth stages to determine the status of the current temperature, relative humidity, air velocity, and ${\mathrm{CO}}_{2}$ concentration in the broiler house. Combined with fixed-point monitoring data, the system further analyzed environmental distribution differences along the front–rear and left–right directions of the broiler house, thereby enabling dynamic monitoring and visual display of environmental changes in the broiler house. When collecting environmental parameters, the inspection robot synchronously recorded the cage number and channel number to mark the spatial source of the real-time monitoring data. When a monitored value exceeded the suitable range corresponding to the relevant age stage, the system generated a real-time monitoring alarm and retained the location information corresponding to the abnormal data.

According to the environmental requirements of broilers at different growth stages, suitable ranges were defined for temperature, relative humidity, air velocity, and ${\mathrm{CO}}_{2}$ concentration [30,31]. Specifically, the threshold values for relative humidity and ${\mathrm{CO}}_{2}$ concentration are listed in Table 2, the temperature thresholds are listed in Table 3, and the air velocity thresholds are listed in Table 4. When a monitored value fell within the suitable range, the corresponding environmental parameter was judged to be in a normal state; when the monitored value was below the lower limit or above the upper limit, it was judged to be abnormal. Let the monitored value of an environmental parameter at time t be x(t), and let the lower and upper limits corresponding to the broiler age at time t be $L(a_{t})$ and $U(a_{t})$, respectively, where $a_{t}$ denotes the broiler age corresponding to time t. Then, the environmental state determination rule can be expressed as

(18) $$S(t)=\left\{\begin{array}{ll} \mathrm{Low}, & x(t)<L(a_{t})\\ \mathrm{Normal}, & L(a_{t})\leq x(t)\leq U(a_{t})\\ \mathrm{High}, & x(t)>U(a_{t}) \end{array}\right.$$

where S(t) denotes the environmental state label at time t. For ${\mathrm{CO}}_{2}$ concentration, only an upper threshold was defined; when the monitored value exceeded the suitable upper limit, the state was determined as ${\mathrm{CO}}_{2}$ over-limit. Based on the above rules, the system was able to perform real-time state identification of the broiler house environment and provide basic support for subsequent early warning generation and environmental regulation decision-making.

### 2.5. Real-Time Rolling Prediction and Dual-Indicator Early Warning Strategy

To meet the requirements for online monitoring and early intervention of environmental anomalies in broiler houses, this study developed a real-time rolling prediction and dual-indicator early-warning strategy for temperature deviation (TD) and temperature–humidity index (THI). This strategy was based on the environmental time series collected by the inspection robot along a preset route. The raw 3 s inspection data were aggregated using a 5 min time window, and the aggregated sequences were used as model inputs. During each rolling prediction process, the model used the historical information from the most recent 36 time steps to predict TD and THI over the next 30 min. The prediction results were used to characterize the short-term variation trend of the thermal environment in the broiler house. On this basis, this study combined the prediction results of TD and THI to establish dual-indicator early-warning rules, generating warning information from two perspectives: environmental control deviation and comprehensive thermal environmental risk. This strategy can assist farm managers in promptly assessing abnormal thermal environmental conditions in the broiler house in combination with field observations of flock status, such as feed intake, water intake, activity level, and respiratory condition, and in implementing control measures such as ventilation, heating, or cooling.

During real-time operation, multi-source environmental data collected by the inspection robot, indoor fixed monitoring points, and the outdoor monitoring point were first aligned according to their timestamps and then resampled and aggregated into an equally spaced 5 min time series. At any time step t, an input sequence was constructed based on the most recent 36 historical time steps and fed into the trained Wavelet–ECA–GRU models to obtain the predicted TD and THI values at 6 time steps ahead, i.e.,

(19) $${\hat{y}}_{t+H}^{\mathrm{TD}}=f_{\mathrm{TD}}\left(X_{t}^{\mathrm{TD}}\right),\;{\hat{y}}_{t+H}^{\mathrm{THI}}=f_{\mathrm{THI}}\left(X_{t}^{\mathrm{THI}}\right)$$

where $f_{\mathrm{TD}}$ and $f_{\mathrm{THI}}$ denote the TD prediction model and the *THI* prediction model, respectively, and ${\hat{y}}_{t+H}^{\mathrm{TD}}$ and ${\hat{y}}_{t+H}^{\mathrm{THI}}$ denote the predicted values of temperature deviation and temperature–humidity index at the future time point, respectively. During online deployment, the input window was continuously updated forward by one time step, thereby forming a rolling prediction process consistent with the actual sampling interval. The rolling prediction was implemented at the broiler-house level using the aggregated 5 min time series. Accordingly, each prediction update provided a short-term estimate of the overall environmental trend and warning status of the monitored house, rather than a location-specific prediction for each spatial point within the house. During online deployment, a new prediction was generated whenever a new 5 min aggregated sample became available. Practical tests showed that approximately 0.5 s was required to complete model input construction, prediction calculation, threshold judgment, and warning-level output after each new aggregated sample was generated. This processing time was considerably shorter than the 5 min rolling update interval and therefore did not affect the continuous updating of warning results.

Based on the rolling prediction results, threshold judgment and risk warning were performed separately for TD and THI. Specifically, the TD prediction results were used to identify whether there would be a risk of cold deviation or heat deviation in environmental control in the broiler house, whereas the THI prediction results were used to identify whether there would be a humid-heat environmental risk. The TD warning threshold was set with reference to the requirements for ventilation and temperature control in broiler houses reported in Reference [32], which indicated that, during transition ventilation and tunnel ventilation, the indoor temperature should not deviate from the target temperature by more than ±2.5 °C. Therefore, when the predicted TD value was greater than 2.5 °C, a potential heat deviation risk was identified; when the predicted TD value was lower than −2.5 °C, a potential cold deviation risk was identified; otherwise, the condition was judged as normal. For THI, this study referred to the THI-based thermal environmental risk classification reported by temperature and humidity [2], in which THI values were classified into comfort, alert, danger, and emergency categories. To facilitate continuous threshold judgment in the proposed early-warning system, the corresponding intervals were expressed as THI < 70, 70 ≤ THI < 76, 76 ≤ THI < 81, and THI ≥ 81, respectively. Kim et al. [2] also showed that higher THI conditions were associated with decreased feed intake and body weight gain, as well as increased respiratory rate and rectal temperature, indicating that THI is closely related to physiological responses and production performance changes caused by heat stress in broilers. The specific classification criteria are shown in Table 5.

On this basis, the system separately generated warning information according to the rolling prediction results of TD and THI. When the predicted TD was lower than the cold-deviation threshold or higher than the heat-deviation threshold, the system issued a cold-deviation warning or a heat-deviation warning, respectively. When the predicted THI reached the alert level or above, the system issued a humid-heat risk warning at the corresponding level. If the warning conditions for both TD and THI were met simultaneously, the system issued both types of warning information at the same time. In this way, the proposed strategy can identify environmental anomalies in broiler houses in advance from two dimensions: environmental control deviation and comprehensive thermal environmental risk, and can support more targeted regulation measures such as ventilation, heating, and cooling.

## 3. Results and Discussion

### 3.1. Correlation Analysis and Feature Selection (Pearson)

In studies on environmental prediction in livestock and poultry houses, Pearson correlation analysis is commonly used for the preliminary screening of input variables to reduce redundant variables and improve the relevance of model inputs. For example, previous studies on apparent temperature prediction in broiler houses have used Pearson correlation coefficients to screen input parameters [8]. Studies on environmental parameter prediction in pig houses have also used Pearson correlation analysis to evaluate the correlations between candidate environmental variables and prediction targets, and to determine model input variables accordingly [22]. Therefore, in this study, Pearson correlation coefficients were used to conduct a preliminary analysis of the linear correlations between candidate variables and TD and THI.

**Figure 6:**
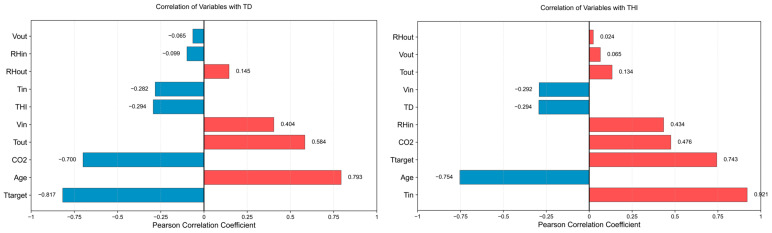
Pearson correlation coefficients between candidate variables and TD and THI.

In this study, the Pearson correlation coefficient (PCC) was used for correlation analysis. The value range of the Pearson correlation coefficient r is $\left[-1,1\right]$, where r>0 indicates a positive correlation between variables, r<0 indicates a negative correlation, and a larger ∣r∣ indicates a stronger linear correlation between variables. To quantitatively interpret the degree of correlation, the correlations were classified according to the absolute value of the Pearson correlation coefficient. When ∣r∣<0.30, the correlation between variables was considered negligible; when 0.30≤∣r∣<0.50, a low correlation was considered to exist; when 0.50≤∣r∣<0.70, a moderate correlation was considered to exist; when 0.70≤∣r∣<0.90, a high correlation was considered to exist; and when ∣r∣≥0.90, a very high correlation was considered to exist [33]. The Pearson correlation coefficient was calculated as follows:

(20) $$r=\frac{\sum_{i=1}^{n}\left(x_{i}-\bar{x})(y_{i}-\bar{y}\right)}{\sqrt{\sum_{i=1}^{n}{\left(x_{i}-\bar{x}\right)}^{2}\sum_{i=1}^{n}{\left(y_{i}-\bar{y}\right)}^{2}}}$$

where $x_{i}$ and $y_{i}$ denote the observed values of variables x and y at the i-th sample, respectively; $\overset{ˉ}{x}$ and $\overset{ˉ}{y}$ denote the sample means of the corresponding variables, respectively; and n denotes the number of samples. Table 6 lists the names, meanings, and units of the main parameters involved in the correlation analysis and prediction modeling in this study, thereby providing a unified description for subsequent variable screening and model input design. Figure 6 shows the Pearson correlation coefficients between temperature deviation (TD), temperature–humidity index (THI), and each candidate variable. In the figure, red indicates positive correlations, blue indicates negative correlations, and the values beside the bars represent the Pearson correlation coefficients between the corresponding variables and the target variable. The results show that the absolute Pearson correlation coefficients between some candidate variables and TD or THI were lower than 0.50, indicating weak linear correlations with the target variables and relatively limited direct contributions to the prediction results. In contrast, Age, $T_{\mathrm{out}}$, ${\mathrm{CO}}_{2}$ and $T_{\mathrm{target}}$ showed moderate or stronger correlations with TD and were therefore selected as input features for the TD prediction model. For THI, Age, $T_{\mathrm{in}}$ and $T_{\mathrm{target}}$ also showed moderate or stronger correlations with the target variable and were therefore included as inputs to the THI prediction model. The input parameters of the TD and THI prediction models are shown in Table 7.

### 3.2. Comparison of Predictive Performance Among Different Models

**Figure 7:**
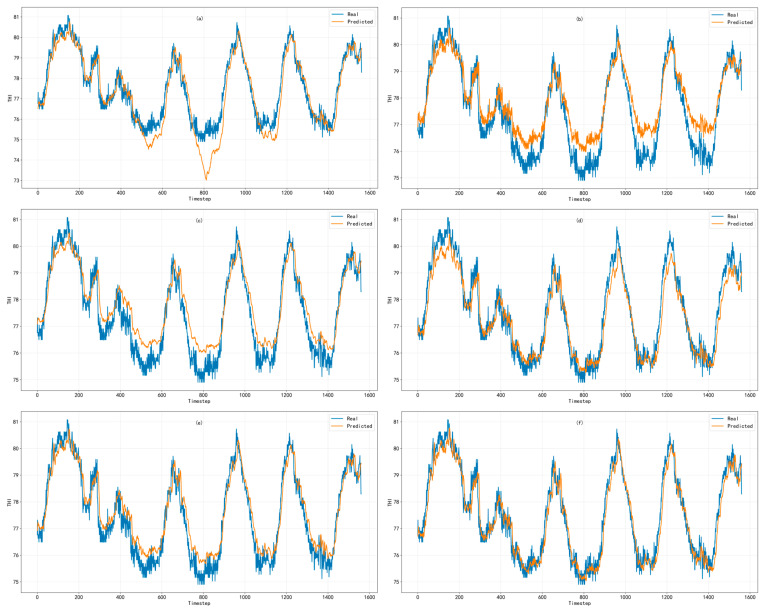
Comparison between observed and predicted values for temperature–humidity index (THI) prediction: (**a**) LSTM, (**b**) TCN, (**c**) GRU, (**d**) ECA–GRU, (**e**) Wavelet–GRU, and (**f**) Wavelet–ECA–GRU.

**Figure 8:**
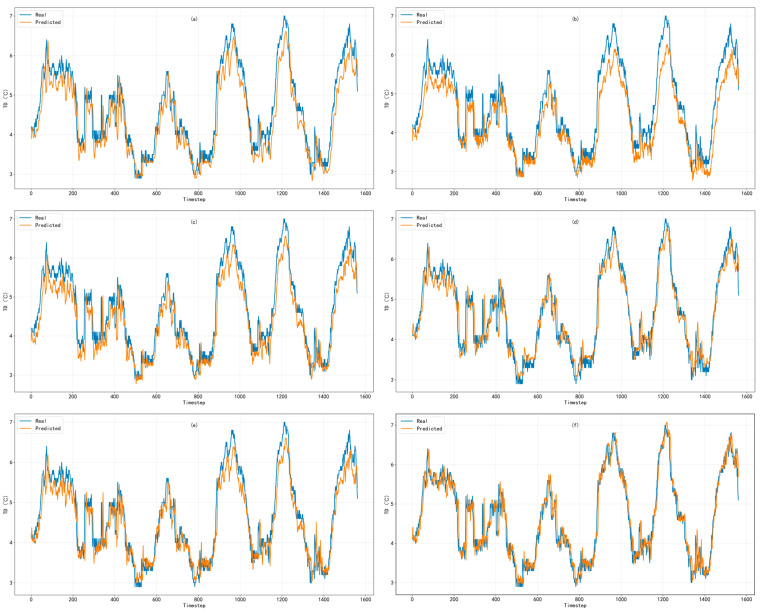
Comparison between observed and predicted values for temperature deviation (TD) prediction: (**a**) LSTM, (**b**) TCN, (**c**) GRU, (**d**) ECA–GRU, (**e**) Wavelet–GRU, and (**f**) Wavelet–ECA–GRU.

To visually demonstrate the temporal representation characteristics of different models in short-term prediction, the same continuous time segment from the test set was selected, and the measured and predicted values for the THI and TD prediction tasks were visualized separately. Figure 7 and Figure 8 show the subfigure-based comparisons of six models for the THI and TD prediction tasks, respectively, where the blue line represents the measured values and the orange line represents the predicted values. The subfigures correspond, from left to right and from top to bottom, to: (a) LSTM, (b) TCN, (c) GRU, (d) ECA–GRU, (e) Wavelet–GRU, and (f) Wavelet–ECA–GRU.

#### 3.2.1. Comparison of THI Prediction Results

As shown in Figure 7, the fitting ability of different models differed considerably in the THI prediction task. Overall, all models were able to reflect the general variation trend of the THI series to some extent; however, their prediction performance differed in intervals involving local rapid fluctuations, abrupt changes, and high-value variations.

As shown in Figure 7a,b, although LSTM and TCN could capture the overall variation direction of THI, obvious amplitude compression and local lag were still observed in intervals with rapid fluctuations or abrupt changes. This indicates that the ability of these two models to respond to non-stationary disturbances remains limited in the current task. One possible reason is that, although LSTM has strong long-term memory capability, its relatively complex gating structure may lead to delayed updating of effective information in short-term high-frequency fluctuation scenarios, thereby weakening the model’s response to rapid changes. TCN mainly relies on fixed convolutional receptive fields to extract local temporal patterns. When a sequence simultaneously contains trend components, periodic variations, and abrupt disturbances, its ability to represent cross-scale dynamic relationships is relatively limited.

By comparison, as shown in Figure 7c, GRU better preserved the peak–valley structure and variation rhythm of the THI series during most periods, indicating that it was more effective in modeling short-term temporal dependencies in the current task. This is related to the relatively compact gated update mechanism of GRU, which can retain necessary historical state information while more directly incorporating changes in current inputs. Therefore, in scenarios with limited sample size and a focus on short-term prediction, GRU is more likely to capture the dynamic variation patterns in broiler house environmental sequences. However, in some high-value fluctuation intervals, the original GRU still showed a certain degree of underestimation, suggesting that when relying only on temporal memory, the model remains limited in recovering amplitudes under the superposition of complex disturbances.

After introducing the ECA mechanism into GRU, as shown in Figure 7d, the model responded more sufficiently in key fluctuation intervals, and the prediction offset near some extreme values was alleviated. By modeling local interactions among channels, ECA strengthened feature representations more relevant to THI variations and reduced the interference of redundant information to some extent, enabling the model to focus more on input information that contributed substantially to the prediction results.

Furthermore, as shown in Figure 7e, Wavelet–GRU tracked abrupt-change intervals and high-frequency disturbances more stably after wavelet decomposition was introduced. The main reason is that wavelet decomposition first decomposes the original THI series into subsequences in different frequency bands, allowing long-term smooth trends and short-term local fluctuations to be separated in the feature space. GRU then learns the temporal dependencies of components at different scales. This processing reduces the difficulty of directly modeling the original mixed signal and weakens the interference of high-frequency disturbances in trend modeling, making it more suitable for handling the non-stationarity and multi-scale fluctuation characteristics commonly present in broiler house environmental sequences.

Overall, as shown in Figure 7f, Wavelet–ECA–GRU achieved better local fitting performance in most key intervals. Its advantages were reflected not only in the accuracy of overall trend fitting, but also in the stable representation of local fluctuations, extreme-value changes, and turning-point characteristics. This improvement mainly benefited from the synergistic effects of wavelet decomposition, the ECA mechanism, and GRU. Wavelet decomposition was used to alleviate the non-stationarity and scale-mixing problems of the input sequence, ECA was used to adaptively enhance key feature channels, and GRU was used to capture temporal dynamic dependencies in the sequence. The combination of these three components effectively improved the representation capability and prediction stability of the model for complex broiler house environmental sequences.

#### 3.2.2. Comparison of TD Prediction Results

Figure 8 shows the performance of each model in the TD prediction task. According to the actual sequence trend, TD gradually increased from a relatively small deviation to a higher level in the early stage. In the middle stage, TD switched several times among different deviation levels and was accompanied by rapid decreases within short periods. In the later stage, several prominent deviation peaks and rapid declining processes appeared. These changes may be related to the accumulation of indoor temperature deviation from the target temperature, the decline after the intervention of control measures, and the combined influence of external environmental fluctuations.

Overall, all models were able to reconstruct the general variation process of the TD sequence. As shown in Figure 8a,b, although LSTM and TCN could reflect the overall variation direction of TD, obvious deviations still occurred in several intervals with large deviations and during stages with rapid changes in deviation. The main limitation of LSTM was not merely local underestimation; rather, it responded slowly during transitions from one stable level to another. This suggests that, while using historical information, LSTM tends to retain the inertia of the previous state, thereby weakening its immediate representation of rapid switching processes. In contrast, the deviation of TCN was more systematic, as it showed a relatively persistent overall overestimation in multiple intervals. This indicates that although the local convolution-based learning mechanism can extract short-term structures, it is insufficient for stably characterizing baseline changes among different state intervals in the TD sequence.

By comparison, as shown in Figure 8c, GRU maintained the variation rhythm of TD more effectively during most periods, indicating that it was more effective in modeling short-term dependencies and state updates. This is related to the relatively compact gated structure of GRU, which can retain necessary historical information while more flexibly incorporating current input changes. Therefore, GRU is more suitable for processing environmental sequences such as TD, which involve frequent state transitions. However, the original GRU still showed some underestimation in certain high-value intervals, suggesting that relying solely on temporal memory remains limited in recovering local extreme values and rapid increasing processes.

On this basis, as shown in Figure 8d,e, ECA–GRU and Wavelet–GRU improved TD prediction from different perspectives. ECA–GRU enhanced key input information through channel recalibration, making the model more sensitive near peaks and in local disturbance intervals; however, local amplification also occurred at some positions with abrupt changes. Wavelet-GRU separated the slowly varying components and rapid short-term fluctuations in the sequence through wavelet decomposition, thereby reducing the interference of complex mixed signals in direct modeling. As a result, it showed more balanced performance in intervals involving increasing deviation, decline, and obvious local fluctuations.

Overall, as shown in Figure 8f, Wavelet–ECA–GRU exhibited more stable prediction performance in key intervals, especially during local rapid fluctuations and state-switching stages, where both amplitude deviation and phase lag were reduced. This improvement may be attributed to the synergistic effect of wavelet decomposition and the ECA mechanism. The former helps separate slow-varying trends and fast-changing disturbances in the sequence, while the latter strengthens the extraction of key feature information, thereby improving the model’s ability to represent complex dynamic processes.

### 3.3. Performance Evaluation of Different Prediction Models

To further quantitatively compare the performance differences among the models in short-term prediction tasks, the prediction results on the test set were evaluated using MSE, MAE, RMSE, and $R^{2}$. The results of the models for THI and TD are presented in Table 8 and Table 9, respectively.

#### 3.3.1. Comparison of THI Prediction Performance

As shown in Table 8, the performance differences among the models in the THI prediction task were relatively obvious, among which the Wavelet–ECA–GRU model achieved the best results. Its MSE, MAE, and RMSE were 0.1857, 0.3326, and 0.4309, respectively, and the $R^{2}$ value reached 0.9287. All of these metrics were better than those of the other comparison models, indicating that this model had higher prediction accuracy and stronger fitting ability for THI prediction.

Compared with the baseline GRU model, the MSE, MAE, and RMSE of the Wavelet–ECA–GRU model decreased by 49.26%, 34.32%, and 28.78%, respectively, while $R^{2}$ increased from 0.8594 to 0.9287. This indicates that, in the THI prediction task, wavelet decomposition and the ECA mechanism could also form an effective complementary combination, thereby enhancing the model’s ability to represent complex temporal features. Both ECA–GRU and Wavelet–GRU also outperformed the baseline GRU. Specifically, for ECA–GRU, MSE, MAE, and RMSE decreased by 34.62%, 23.64%, and 19.14%, respectively, and $R^{2}$ increased to 0.9081. For Wavelet–GRU, the three error metrics decreased by 32.62%, 19.99%, and 17.92%, respectively, and $R^{2}$ increased to 0.9053. These results indicate that introducing either the attention mechanism or wavelet decomposition alone could improve THI prediction performance, while their combined use produced a more pronounced improvement.

From the results of the baseline models, LSTM and TCN showed relatively higher overall errors and lower $R^{2}$ values, indicating that these models still had certain limitations in handling THI series characterized by non-stationarity and complex fluctuations. By contrast, the improved GRU-based models were better able to adapt to the multi-scale variations and key feature differences in the THI series, thereby exhibiting better predictive performance.

Overall, the Wavelet–ECA–GRU model achieved the best results in both TD and THI prediction tasks, indicating that the combination of wavelet decomposition and the ECA mechanism can effectively improve the short-term prediction capability of the GRU model for broiler house environmental series.

#### 3.3.2. Comparison of TD Prediction Performance

As shown in Table 9, the performance differences among the models in the TD prediction task were relatively evident. Overall, the GRU-based models outperformed LSTM and TCN, among which the Wavelet–ECA–GRU model achieved the best prediction results. Its MSE, MAE, and RMSE were 0.0833, 0.2077, and 0.2886, respectively, and the $R^{2}$ value reached 0.9238. These results indicate that the model was able to more accurately capture the variation process of the TD series, showing advantages in both error control and fitting ability.

Using the baseline GRU as a reference, the MSE, MAE, and RMSE of Wavelet–ECA–GRU decreased by 48.83%, 36.27%, and 28.48%, respectively, while $R^{2}$ increased from 0.8510 to 0.9238. This indicates that simultaneously introducing wavelet decomposition and the ECA mechanism into the GRU model significantly improved its ability to model the TD series. Meanwhile, both ECA–GRU and Wavelet–GRU also performed better than the baseline GRU. Specifically, for ECA–GRU, MSE, MAE, and RMSE decreased by 20.95%, 18.59%, and 11.10%, respectively, and $R^{2}$ increased to 0.8822. For Wavelet–GRU, the three error metrics decreased by 25.31%, 16.97%, and 13.58%, respectively, and $R^{2}$ increased to 0.8887. These results indicate that either introducing the attention mechanism or incorporating multi-scale decomposition features could improve TD prediction accuracy, while the combined use of both produced a more pronounced improvement.

By contrast, the overall performance of LSTM and TCN was relatively weaker. In particular, TCN performed worse than the GRU-based models on all four metrics, indicating that its ability to capture the local fluctuations and temporal dependency characteristics of the TD series in this study was insufficient. Overall, the Wavelet–ECA–GRU model achieved the best performance in both the THI and TD prediction tasks, indicating that simultaneously introducing wavelet decomposition and the ECA mechanism into the GRU model can further enhance its short-term prediction capability for broiler house environmental series.

Considering the results of both the THI and TD prediction tasks, Wavelet–ECA–GRU achieved the best performance among the comparison models established in this study. This indicates that the advantage of the proposed model was not reflected only in a single error metric, but also in multiple aspects, including overall fitting ability, average error control, and suppression of large deviations. These results suggest that the combination of wavelet decomposition, the ECA mechanism, and GRU can improve the short-term prediction capability of broiler house environmental sequences at different levels. Specifically, GRU captures temporal dynamic dependencies, wavelet decomposition helps alleviate the interference caused by non-stationarity, multi-scale fluctuations, and local high-frequency disturbances in the original environmental sequences, and the ECA mechanism further enhances the extraction of key feature information. These three components are not simply superimposed; rather, they form strong complementary effects in terms of input representation, key information enhancement, and temporal modeling. Therefore, the combined model outperformed the models that introduced only one of these improvement strategies. Previous studies have indicated that sequence decomposition methods can improve the representation of complex environmental time-series characteristics by extracting variation information at different scales [18]. In addition, deep-learning and hybrid prediction models have also been applied to multi-parameter environmental prediction in poultry and pig houses [19,20,21,22]. In line with these methodological trends, the improved performance of the Wavelet–ECA–GRU model in this study may be attributed to the complementary effects of multi-scale decomposition, the channel attention mechanism, and GRU-based temporal modeling. However, unlike studies that mainly focus on a single environmental variable or a single comprehensive thermal index, this study further links the short-term prediction results with TD- and THI-based warning information, thereby extending model comparison to decision-oriented environmental management in caged broiler houses.

From the differences between the THI and TD prediction results, the improved models showed a consistent performance improvement trend in both tasks, indicating that the proposed method has a certain degree of stability and is not effective only for a specific indicator. Meanwhile, the magnitudes of improvement differed between the two tasks, suggesting that THI and TD place different emphases on model capability. As a comprehensive index formed by the coupling of temperature and humidity, THI is related not only to the numerical state of the environment itself, but also closely to the thermal comfort level of broilers. Previous studies have shown that when THI or heat load increases, broilers usually dissipate heat by increasing respiratory rate, spreading their wings, increasing water intake, and reducing activity. If high-temperature and high-humidity conditions persist, they may further lead to reduced feed intake, elevated body temperature, impaired growth performance, and decreased overall comfort [2]. Therefore, when the model predicts that THI will continue to increase or remain at a high level, farm managers should promptly check and adjust ventilation, cooling, and water supply measures in combination with observed heat-stress behaviors of the flock, so as to reduce the adverse effects of hot and humid environments on broiler comfort and production performance.

In comparison, TD more directly reflects the degree to which the actual temperature deviates from the target temperature, and therefore can characterize dynamic deviations during environmental regulation in broiler houses. A continuous increase in TD indicates that the broiler house temperature is gradually becoming higher than the target level. This is usually associated with insufficient ventilation, low air velocity, or limited cooling effectiveness, which weakens the ability of broilers to dissipate heat through convection and evaporation. Under such conditions, the flock is more likely to be exposed to a heat-deviation environment and may gradually show responses such as increased panting, more frequent lying behavior, reduced feed intake, and decreased activity [34]. When TD continues to decrease and the actual temperature becomes lower than the target temperature, it may indicate that the flock is exposed to a cold-deviation condition. This is usually related to insufficient heating or inappropriate ventilation strategies. Previous studies have shown that cold stress can adversely affect broiler health and welfare and may induce immune imbalance, reduced antioxidant capacity, and physiological disorders. Behaviorally, broilers usually reduce heat loss by huddling, curling up, and decreasing activity to maintain body temperature stability [35]. Accurate prediction of local abrupt changes, state-transition intervals, and short-term rapid fluctuations in TD can be used to determine whether the environmental control system in the broiler house deviates from the set state. When TD continues to increase, the operation of the ventilation and cooling systems should be checked first. When TD continues to decrease and falls below the target temperature, heating and ventilation strategies should be adjusted in time, thereby reducing the adverse effects of heat and cold stress on broiler health and welfare.

It should be noted that the model performance evaluation results in this study are still limited by the data source, experimental scenario, and sample coverage. First, the data used in this study were obtained from a single production cycle and a single broiler house facility. Although this allowed the prediction performance of the model to be verified under relatively consistent environmental conditions, it also limited the applicability of the findings to broader production conditions. Under different environmental conditions, the patterns of temperature and humidity variation inside broiler houses may differ substantially. Previous studies have shown that seasonal conditions, broiler house structure, and ventilation mode can affect indoor airflow as well as the distribution characteristics of temperature and humidity [36,37]. In addition, differences in stocking density and ventilation control level may further alter the indoor thermal environment and environmental regulation load. These factors may change the distribution range, fluctuation amplitude, and state-transition characteristics of TD and THI, thereby affecting the stability and applicability of the model in different production scenarios. Therefore, further validation under different production cycles, seasons, and broiler house conditions is still needed in future studies to more comprehensively evaluate the practical application performance of the model in commercial production.

### 3.4. Effect of Wavelet Basis Functions on Prediction Performance

To further analyze the effect of wavelet basis function selection on model prediction performance, three commonly used wavelet bases, namely db2, db4, and sym4, were selected for comparative experiments while keeping all other model parameters unchanged. The prediction results of the model for TD and THI under different wavelet basis functions are presented in Table 10.

As shown in Table 10, the selection of the wavelet basis function had a certain influence on the prediction accuracy of the model. For the TD prediction task, the model using the db4 wavelet basis achieved the lowest MAE and RMSE values of 0.2077 and 0.2886, respectively, while obtaining the highest $R^{2}$ value of 0.9238. Compared with db2 and sym4, db4 showed superior performance in terms of error control and goodness of fit. For the THI prediction task, db4 also achieved the best prediction performance, with MAE, RMSE, and $R^{2}$ values of 0.3326, 0.4309, and 0.9287, respectively, outperforming the other two wavelet basis functions.

Considering the MAE, RMSE, and $R^{2}$ metrics for both TD and THI prediction tasks, the db4 wavelet basis exhibited the best overall performance among the three wavelet basis functions, enabling higher prediction accuracy and better fitting performance in the current prediction tasks. Therefore, db4 was selected as the wavelet basis function for subsequent model construction.

### 3.5. Analysis of Early Warning Results

**Figure 9:**
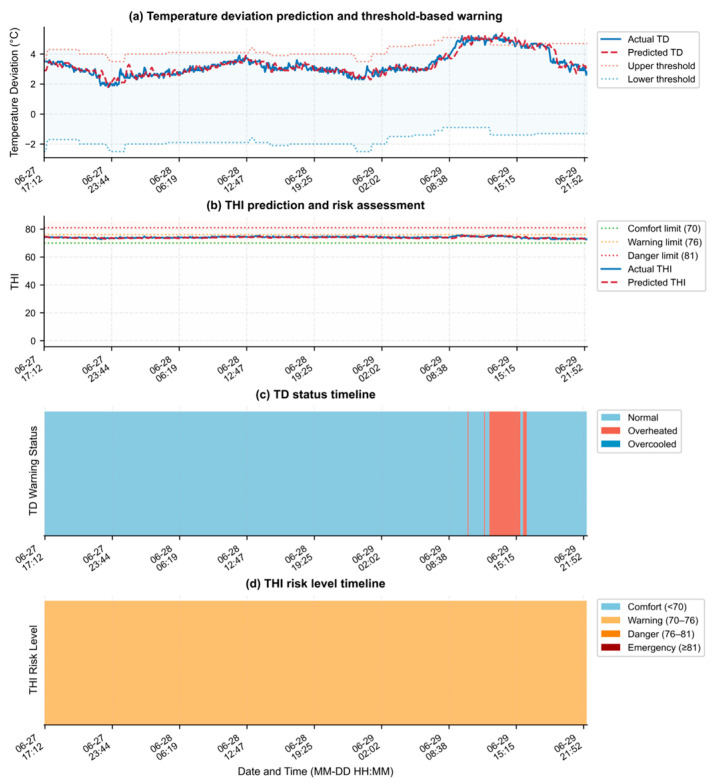
Rolling prediction and early warning results of TD and THI during the test period.

To evaluate the application performance of the proposed dual-indicator early warning method during practical operation, the rolling prediction results of TD and THI, together with the corresponding warning states during the test period, were visualized and analyzed in combination with statistical results, as shown in Figure 9 and Table 11. As can be seen from Figure 9a,c, the predicted TD curve was generally consistent with the actual curve in terms of the overall variation trend, and it was able to reflect the dynamic variation characteristics of temperature deviation in the broiler house during the test period. In particular, during overheating periods, the prediction results were able to track the actual fluctuations relatively well, and the corresponding warning-state timeline was largely consistent with the actual warning results. Table 11 further shows that, among the 596 test samples, the actual TD states were dominated by the normal state, whereas the overheating state accounted for a relatively small proportion, and the predicted distribution was generally consistent with the actual distribution. Specifically, the actual and predicted proportions of the normal state were 93.62% and 93.29%, respectively, while those of the overheating state were 6.38% and 6.71%, respectively. Meanwhile, the deviation in overheating frequency was 5.26%, and the consistency of state distribution reached 99.66%. This indicates that, under the current test conditions, the model was able to characterize the overall distribution pattern of temperature deviation in the broiler house and showed good consistency with the classification results for the small number of heat-deviation cases during the test period. It should be noted that, because heat-deviation samples accounted for only a small proportion of the dataset, the current results are still insufficient to fully validate the model’s ability to identify overheating under frequent, persistent, or more severe temperature-deviation conditions. Further validation is therefore needed under different seasons, broiler house facilities, stocking densities, and ventilation control conditions to evaluate the model’s ability to identify TD states.

As shown in Figure 9b,d, the predicted THI curve was also generally consistent with the actual curve, and all test samples fell within the warning level. Table 11 shows that, for the 596 samples, both the actual and predicted classifications were at the warning level, resulting in a classification distribution consistency of 100%. It should be emphasized that, because all THI classifications corresponding to the current test samples were at the alert level, with no samples falling into the comfort, danger, or emergency levels, the 100% consistency rate in classification distribution shown in Table 11 only indicates that the model prediction results were consistent with the threshold-based judgment under the current single risk level. It does not demonstrate that the model already has the ability to identify multi-level THI risks.

Considering the TD and THI results together, these two indicators describe the environmental status of the broiler house from the perspectives of temperature deviation and temperature–humidity coupled thermal load, respectively, and therefore show a certain degree of complementarity. Specifically, TD reflects the degree to which the actual temperature deviates from the target temperature, whereas THI characterizes the thermal environmental risk level under the combined effects of temperature and humidity. Based on the current test results, the dual-indicator early-warning method can reflect changes in the environmental status corresponding to TD and THI during the test period. Given that heat-deviation events accounted for only a small proportion of the current TD test samples and that the THI samples were concentrated within a single alert level, future studies will further conduct tests under different seasons, broiler house facilities, stocking densities, and ventilation control conditions to evaluate the application performance of this method under more severe temperature-deviation conditions and multi-level THI risk scenarios.

## 4. Conclusions

This study addressed the practical requirements for online environmental monitoring and risk warning in caged broiler houses by developing a multi-source environmental monitoring and short-term early-warning framework integrating an inspection robot, fixed indoor monitoring points, and an outdoor monitoring point. For the two indicators, temperature deviation (TD) and temperature–humidity index (THI), 30 min rolling prediction models based on Wavelet–ECA–GRU were established separately, and a dual-indicator early-warning strategy was designed to identify environmental anomalies in broiler houses from two perspectives: environmental regulation deviation and temperature–humidity coupled thermal risk.

The results showed that, under the data conditions of this study, Wavelet–ECA–GRU achieved favorable prediction performance in both the TD and THI prediction tasks, outperforming the comparison models, including LSTM, TCN, GRU, ECA–GRU, and Wavelet–GRU. Specifically, the MSE, MAE, RMSE, and R^2^ values for TD prediction were 0.0833, 0.2077, 0.2886, and 0.9238, respectively, while those for THI prediction were 0.1857, 0.3326, 0.4309, and 0.9287, respectively. These results indicate that the integration of multi-scale decomposition, channel attention, and GRU-based temporal modeling can effectively characterize short-term fluctuations, local disturbances, and variation trends in broiler house environmental sequences.

From the perspective of practical application, TD and THI reflect different aspects of thermal environmental management in broiler houses. TD directly characterizes the degree to which the actual temperature deviates from the target temperature and can be used to identify potential heat-deviation or cold-deviation risks during environmental control. THI, in contrast, comprehensively reflects the thermal environmental load under the combined effects of temperature and humidity and can be used to identify humid-heat risk. The combination of these two indicators can reduce the limitations associated with relying only on a single temperature variable or a single comprehensive thermal environmental index, allowing the warning results to cover both environmental control deviation and temperature–humidity coupled risk. In practical production, this method can alert farm managers in advance to changes in the thermal environment of broiler houses and help them adjust ventilation rate, heating intensity, and cooling measures in a timely manner in combination with field observations of flock status, such as feed intake, water intake, activity level, and respiratory condition.

Further analysis of the warning results showed that, during the current test period, the TD-based warning results were relatively close to the actual state distribution and were able to reflect changes in environmental control deviation in the broiler house. For THI-based warnings, because the test samples were mainly concentrated at the warning level, the model prediction results were consistent with the threshold-based judgment under the current single risk level. However, its identification capability in multi-level risk scenarios still requires further validation. These results indicate that the TD and THI dual-indicator prediction and early-warning method based on multi-source environmental data has certain practical feasibility and can serve as an auxiliary approach for the early identification of environmental anomalies in caged broiler houses.

Nevertheless, this study still has several limitations. The data were collected from a single broiler house, a single batch, and one production cycle, resulting in relatively limited scenario coverage. The distribution of risk levels in the warning test samples was also imbalanced. In particular, the THI samples did not cover additional risk levels such as comfort, danger, and emergency; therefore, the current results are not sufficient to fully demonstrate the model’s comprehensive identification capability under complex environmental conditions and multi-level risk scenarios. Future studies will incorporate multi-season, multi-house, and multi-batch data to further validate the generalization ability and warning stability of the model, and to optimize the applicability of thresholds and warning strategies under different environmental conditions.

## Figures and Tables

**Table 1:** Relevant parameters of the environmental sensors.

Sensor Index	Unit	Range	Accuracy	Sensor Model
Temperature	°C	−20~+50 °C	±0.2 °C	JES-CO2TR-AR1-C2P0
Humidity	%	0~100% RH	±2% RH	JES-CO2TR-AR1-C2P0
Wind speed	m/s	0.1~5 m/s	±(0.2 m/s ± 0.02 × v)	JES-WS-CR1-C2P0
Carbon dioxide	ppm	300~5000 ppm	±50 ppm	JES-CO2TR-AR1-C2P0

**Table 2:** Suitable ranges of relative humidity and ${\mathrm{CO}}_{2}$ concentration at different broiler ages.

Age (Days)	Suitable Range of Humidity	Suitable CO_2_ Concentration Range
1–10	65–70%	${\mathrm{CO}}_{2}\leq 1500\;\mathrm{ppm}$
11–30	60–65%	${\mathrm{CO}}_{2}\leq 1500\;\mathrm{ppm}$
31–45	55–60%	${\mathrm{CO}}_{2}\leq 1500\;\mathrm{ppm}$
46–	50–55%	${\mathrm{CO}}_{2}\leq 1500\;\mathrm{ppm}$

**Table 3:** Suitable temperature ranges at different broiler ages.

Age (Days)	Suitable Temperature Range
1–3	34–35 °C
4–7	32–33 °C
8–15	30–31 °C
16–23	28–29 °C
24–31	26–27 °C
32–39	24–25 °C
40–	22–23 °C

**Table 4:** Suitable air velocity ranges at different broiler ages.

Age (Days)	Suitable Air Velocity Range
1–14	0–0.20 m/s
15–21	0–0.51 m/s
22–28	0–1.02 m/s
29–	1.5–2.5 m/s

**Table 5:** Thermal environmental risk classification based on the temperature–humidity index (THI).

THI Range	Thermal Environment Status
THI<70	Comfort
70≤THI<76	Warning
76≤THI<81	Danger
THI≥81	Emergency

**Table 6:** Description and units of the main variables used in correlation analysis and prediction modeling.

Parameter	Description	Unit
Age	Age of broiler chickens	d
TD	Temperature deviation	°C
THI	Temperature–Humidity Index	-
${\mathrm{CO}}_{2}$	${\mathrm{Indoor}\;\mathrm{CO}}_{2}\;\mathrm{concentration}$	ppm
$T_{\mathrm{in}}$	Indoor temperature	°C
$T_{\mathrm{target}}$	Target temperature	°C
$T_{\mathrm{out}}$	Outdoor temperature	°C
${\mathrm{RH}}_{\mathrm{in}}$	Indoor relative humidity	%
${\mathrm{RH}}_{\mathrm{out}}$	Outdoor relative humidity	%
$V_{\mathrm{out}}$	Outdoor wind speed	m/s
$V_{\mathrm{in}}$	Indoor wind speed	m/s

**Table 7:** Input features of the TD and THI prediction models.

Prediction Target	Input Parameters
TD	Age, TD, $T_{\mathrm{out}}$, ${\mathrm{CO}}_{2}$, $T_{\mathrm{target}}$
THI	$\mathrm{Age},\;\mathrm{THI},\;T_{\mathrm{in}}$, $T_{\mathrm{target}}$

**Table 8:** Performance comparison of different models for temperature–humidity index (THI) prediction.

Model	MSE	MAE	RMSE	$R^{2}$
LSTM	0.4341	0.4955	0.6588	0.8332
TCN	0.4829	0.5687	0.6949	0.8144
GRU	0.3660	0.5064	0.6050	0.8594
ECA-GRU	0.2393	0.3867	0.4892	0.9081
Wavelet-GRU	0.2466	0.4052	0.4966	0.9053
Wavelet-ECA-GRU	0.1857	0.3326	0.4309	0.9287

**Table 9:** Performance comparison of different models for temperature deviation (TD) prediction.

Model	MSE	MAE	RMSE	$R^{2}$
LSTM	0.1714	0.3297	0.4140	0.8431
TCN	0.2154	0.3765	0.4641	0.8029
GRU	0.1628	0.3259	0.4035	0.8510
ECA-GRU	0.1287	0.2653	0.3587	0.8822
Wavelet-GRU	0.1216	0.2706	0.3487	0.8887
Wavelet-ECA-GRU	0.0833	0.2077	0.2886	0.9238

**Table 10:** Effect of different wavelet basis functions on prediction performance.

Prediction Target	Wavelet Basis	MAE	RMSE	$R^{2}$
TD	db2	0.2399	0.3173	0.9073
TD	db4	0.2077	0.2886	0.9238
TD	sym4	0.2226	0.3057	0.9139
THI	db2	0.3681	0.4730	0.9118
THI	db4	0.3326	0.4309	0.9287
THI	sym4	0.3636	0.4638	0.9152

**Table 11:** Statistical analysis results of TD and THI early warnings.

Metric	Result
TD normal state proportion (Actual/Predicted)	93.62%/93.29%
TD overheating state proportion (Actual/Predicted)	6.38%/6.71%
TD overheating frequency deviation	5.26%
TD state distribution consistency	99.66%
THI warning level proportion (Actual/Predicted)	100%/100%
THI classification distribution consistency	100%

## Data Availability

The data are not publicly available at this time due to ongoing related research but are available from the corresponding author upon reasonable request.
